# Epithelial talin-1 protects mice from *citrobacter rodentium*-induced colitis by restricting bacterial crypt intrusion and enhancing t cell immunity

**DOI:** 10.1080/19490976.2023.2192623

**Published:** 2023-03-23

**Authors:** Yvonne L. Latour, Margaret M. Allaman, Daniel P. Barry, Thaddeus M. Smith, Kamery J. Williams, Kara M. McNamara, Justin Jacobse, Jeremy A. Goettel, Alberto G. Delgado, M. Blanca Piazuelo, Shilin Zhao, Alain P. Gobert, Keith T. Wilson

**Affiliations:** a Department of Pathology, Microbiology, and Immunology, Vanderbilt University Medical Center, Nashville, TN, USA; b Division of Gastroenterology, Hepatology, and Nutrition, Department of Medicine, Vanderbilt University Medical Center, Nashville, TN, USA; c Program in Cancer Biology, Vanderbilt University School of Medicine, Nashville, TN, USA; d Center for Mucosal Inflammation and Cancer, Vanderbilt University Medical Center, Nashville, TN, USA; e Department of Biostatistics, Vanderbilt University Medical Center, Nashville, TN, USA; f Medical Service, Veterans Affairs Tennessee Valley Healthcare System, Nashville, TN, USA

**Keywords:** Talin, C. rodentium, colonic epithelial cells, neutrophils, T cells, colon organoids, fecal microbiome, flow cytometry, crypt hyperplasia

## Abstract

Pathogenic enteric *Escherichia coli* present a significant burden to global health. Food-borne enteropathogenic *E. coli* (EPEC) and Shiga toxin-producing *E. coli* (STEC) utilize attaching and effacing (A/E) lesions and actin-dense pedestal formation to colonize the gastrointestinal tract. Talin-1 is a large structural protein that links the actin cytoskeleton to the extracellular matrix though direct influence on integrins. Here we show that mice lacking talin-1 in intestinal epithelial cells (*Tln1*^*Δepi*^) have heightened susceptibility to colonic disease caused by the A/E murine pathogen *Citrobacter rodentium*. *Tln1*^*Δepi*^ mice exhibit decreased survival, and increased colonization, colon weight, and histologic colitis compared to littermate *Tln1*^*fl/fl*^ controls. These findings were associated with decreased actin polymerization and increased infiltration of innate myeloperoxidase-expressing immune cells, confirmed as neutrophils by flow cytometry, but more bacterial dissemination deep into colonic crypts. Further evaluation of the immune population recruited to the mucosa in response to *C. rodentium* revealed that loss of *Tln1* in colonic epithelial cells (CECs) results in impaired recruitment and activation of T cells. *C. rodentium* infection-induced colonic mucosal hyperplasia was exacerbated in *Tln1*^*Δepi*^ mice compared to littermate controls. We demonstrate that this is associated with decreased CEC apoptosis and crowding of proliferating cells in the base of the glands. Taken together, talin-1 expression by CECs is important in the regulation of both epithelial renewal and the inflammatory T cell response in the setting of colitis caused by *C. rodentium*, suggesting that this protein functions in CECs to limit, rather than contribute to the pathogenesis of this enteric infection.

## Introduction

The colonic epithelium plays a pivotal role in the delicate balance between gut homeostasis and disease. Colonic epithelial cells (CECs) line the lumen of the colon and provide the initial barrier between the microbiome and the rest of the body.^1^ *Escherichia coli*, a prominent member of the human colonic microbiota, usually maintains a commensal relationship with the host, however, enteropathogenic *E. coli* (EPEC) are leading causes of diarrheal-related deaths, especially in children and the elderly.^2,3^ One mode of EPEC pathogenesis is through attaching and effacing (A/E) lesions, a strategy shared by other bacteria, such as Shiga toxin-producing *E. coli* (STEC) and the naturally occurring mouse-restricted pathogen, *Citrobacter rodentium*.^4,5^ The virulence factors that induce A/E lesion formation are encoded by the locus of enterocyte effacement (LEE), which includes genes encoding for a type III secretion system (T3SS) secreted proteins, the adhesin intimin, and its receptor termed translocated intimin receptor (Tir).^4–6^ The formation of pedestal-like structures underneath the intimately adhered bacteria are composed of the bacterial translocated effectors and the recruited host cytoskeletal and focal adhesion proteins α-actinin, vinculin, and talin-1.^6,7^

Talin-1, encoded by the gene *Tln1*, is a ubiquitously expressed mechanosensory scaffold protein that was first discovered in chicken gizzards.^8,9^ The homo-dimeric talin-1 molecule is comprised of two subunits; a 50-kDa N-terminal head containing a FERM domain and a 220-kDa C-terminal rod lined with helical bundles that provide multiple binding sites for actin and vinculin.^9–11^ The family of proteins that possess a FERM domain are often associated with protein-protein interactions that link the cytoskeleton to transmembrane receptors.^12^ The head of talin-1 has been shown to bind to the cytoplasmic domain of the β-subunit of integrins and facilitates a conformational change to the extracellular domain that increases integrin binding affinity.^9,13^ The tail of talin-1 tethers to F-actin and through inside-out signaling, promotes focal adhesion assembly and increases forces exerted on the extracellular matrix (ECM).^14–18^

Increasing evidence suggests that talin-1 is essential for A/E lesion pedestal formation and actin polymerization.^19,20^ We have recently shown that suppression of talin-1 expression in vitro results in decreased actin rearrangement in immortalized young adult mouse colon cells.^21^ Therefore, our aim in this study was to determine the role of talin-1 in CECs during pathogenic enteric bacterial infection in vivo. Cell-specific knockdown of *Tln1* in intestinal epithelial cells (IECs) in mice resulted in increased *C. rodentium* colonization with increased depth of infection in the colonic epithelial glands, associated with decreased actin condensation, enhanced neutrophil infiltration, and impaired T cell response, together resulting in increased clinical and histologic evidence of colitis. In addition, we demonstrate that genetic loss of *Tln1* contributes to colonic crypt hyperplasia. This effect was associated with reduced apoptosis of surface CECs and reduced proliferation along the upper zone of the crypts. Taken together, these findings implicate talin-1 as a regulator of CEC response and T cell recruitment during infectious colitis that restricts *C. rodentium* pathogenesis.

## Materials and methods

### Mice

C57BL/6 *Tln1*^*fl/fl*^ mice were generated by Petrich *et al*. and provided to us by Dr. Roy Zent at Vanderbilt University Medical Center (Nashville, TN).^22,23^ The *Tln1*^*fl/fl*^ mice were then crossed with C57BL/6 *Vil1*^*cre/+*^ mice and the resulting offspring were backcrossed to *Tln1*^*fl/fl*^ mice to generate *Tln1*^*fl/fl*^;*Vil1*^*+/+*^ and *Tln1*^*fl/fl*^;*Vil1*^*cre/+*^ (*Tln1*^*Δepi*^) mice.^24^ The mouse colony was maintained and housed in a specific-pathogen free facility with ventilated cage racks and a 12 h/12 h light/dark cycle. Mice were fed *ad libitum* with 5L0D chow (LabDiet) and provided continuous water. All experiments were approved IACUC at Vanderbilt University and Institutional Biosafety Committee and the Research and Development Committee of the Veterans Affairs Tennessee Valley Healthcare System under the protocol V2000018. Procedures were performed in accordance with institutional policies, AAALAC guidelines, the American Veterinary Medical Association Guidelines on Euthanasia, NIH regulations (Guide for the Care and Use of Laboratory Animals; National Academies Press, 2011), and the US Animal Welfare Act (1966).

### Isolation of colonic epithelial cells

Epithelial cells were isolated from the colonic mucosa as previously described.^21,25^ Briefly, colons were excised, cut longitudinally, washed with PBS, cut into 2 mm pieces, placed in dissociation buffer containing 3 mM DTT and 0.5 mM EDTA, and incubated on ice for one hour. The pieces were then vigorously shaken in PBS and the cells were passed through a 70 μm cell strainer.

### mRNA analysis

Total RNA was isolated from CECs and colonic tissues using the RNeasy Mini Kit (QIAGEN), according to the manufacturer’s instructions. Equal amounts of total RNA were reverse transcribed into cDNA using the SuperScript III Reverse Transcriptase (Thermo Fisher), Oligo (dT) primers (Thermo Fisher), and dNTP Mix (Applied Biosystems). Quantitative real-time PCR was performed using the PowerUp SYBR Green Master Mix (Applied Biosystems) and the primers listed in Table 1.

**Table 1.** List of qRT-PCR primers used for this paper.

Target gene	Sequence (5’−3’)
*Tln1*	F: GGCCCTCCCAACGACTTT
	R: AGCCTCTAGCCAGATGCCTTT
*Ccl5*	F: GGCCATCAGCAACAACATAAGCGT
	R: ACACACTTGGCGGTTCCT
*Ccl20*	F: CGACTGTTGCCTCTCGTACA
	R: AGGAGGTTCACAGCCCTTTT
*Ifng*	F: GGCCATCAGCAACAACATAAGCGT
	R: TGGGTTGTTGACCTCAAACTTGGC
*Il17a*	F: ATCCCTCAAAGCTCAGCGTGTC
	R: GGGTCTTCATTGCGGTGGAGAG
*Il22*	F: TTGAGGTGTCCAACTTCCAGCA
	R: AGCCGGACGTCTGTGTTGTTA
*Tnf*	F: CTGTGAAGGGAATGGGTGTT
	R: GGTCACTGTCCCAGCATCTT
*Actb*	F: CCAGAGCAAGAGAGGTATCC
	R: CTGTGGTGGTGAAGCTGTAG

### Western blot and densitometric analysis

Isolated CECs or colonic tissues were lysed using ice cold CellLytic MT Reagent (Sigma-Aldrich) supplemented with the Protease Inhibitor Cocktail (Set III, Calbiochem) and the Phosphatase Inhibitor Cocktail (Set I, Calbiochem). The BCA Protein Assay (Pierce) was used to measure total protein concentrations. Proteins were separated by SDS-PAGE on a 4–20% gel, transferred to nitrocellulose membranes, and blocked with 5% w/v milk in TBS with 0.1% Tween-20 for 1 h. Membranes were incubated with a rabbit anti-Talin-1 mAb (Cell Signaling, C45F1; 1:2000) overnight at 4°C in 5% w/v BSA in TBS with 0.1% Tween-20 (based on the manufacturer’s recommendations) or a mouse anti-β-actin mAb (Sigma-Aldrich, A1978; 1:10000) in 5% w/v milk in TBS with 0.1% Tween-20 for 30 min at room temperature. Protein bands were visualized by incubating the membrane with HRP-labeled goat anti-rabbit IgG (Jackson ImmunoResearch, 111-035-003; 1:5000) or HRP-labeled goat anti-mouse IgG (Promega, W402B; 1:20000), respectively, and using SuperSignal West Pico PLUS Chemiluminescent Substrate (Pierce) and HyBlot CL Autoradiography Film (labForce). Densitometric analysis of Western blots was performed with Fiji (ImageJ).^26^

### *C. rodentium* Infection

*C. rodentium* strain DBS100 was cultured overnight in Luria-Bertani (LB) broth shaking at 37°C. Adult male *Tln1*^*fl/fl*^ and *Tln1*^*Δepi*^ littermates (6–12 wk) are inoculated by oral gavage with 5 × 10^8^ *C. rodentium* in 0.2 mL LB broth.^21,27,28^ Control mice received 0.2 mL of sterile LB broth alone. Mice were weighed and monitored daily and animals that showed signs of distress, lost more than 20% of initial body weight, or became moribund were euthanized. At 14 days post-inoculation, mice were sacrificed, and the colons were removed, measured, cleaned, weighed, and Swiss-rolled for fixation in 10% neutral buffered formalin and subsequent histology. Three proximal and distal pieces were collected prior to fixation. Two pieces were flash frozen for RNA and protein isolation and analysis and the third was used to determine bacterial colonization by counting the colony forming units (CFUs) after plating serial dilutions of homogenized tissue on McConkey agar plates. *C. rodentium* colonization of the spleen was determined as above.

### Colonic fecal microbiota analysis

Fecal samples from the colon lumen from *Tln1*^*fl/fl*^ and *Tln1*^*Δepi*^ mice were frozen, weighed, and genomic DNA was extracted using the QIAamp Fast DNA Stool Mini Kit (QIAGEN). DNA was quantified using Qubit Fluorometric Quantification (Thermo Fisher Scientific), and the V4 region of the 16S rRNA gene was sequenced with the Illumina MiSeq. Sequences were processed with Mothur, version 1.44.3 (https://mothur.org/), aligned to the SILVA database release 132 (https://www.arb-silva.de/), and taxonomically classified with the Ribosomal Database Project classifier version 16. Nonbacterial sequences and chimeric sequences detected by UCHIME were removed. Operational Taxonomic Unit clustering was performed with VSEARCH, using abundance-based greedy clustering. Rarefaction followed by alpha-diversity, and beta-diversity calculations were repeated 1000 times, and the results were averaged. Data summarization and visualization were performed by R package phyloseq (http://joey711.github.io/phyloseq/).

### Histologic score

Paraffin-embedded Swiss-rolled colons were sectioned (5 μm), stained with hematoxylin (H&E), and examined in a blinded manner by a gastrointestinal pathologist (MBP). The histologic injury score (0–21) is the combination of epithelial injury score assessed on a scale of 0–3 (no injury, mucus depletion, erosion, superficial ulcers or extensive erosion, deep ulcers and/or necrosis of the mucosa) plus total inflammation (0–18), which is the extent of inflammation from 0–3 (no inflammation, mucosa, submucosa, muscular propria or beyond) multiplied by the sum of acute (polymorphonuclear cell infiltration) and chronic (mononuclear cell infiltration) inflammation on a scale of 0–3 for each.^29^

### Immunofluorescence

Immunofluorescent staining for *C. rodentium* was performed on paraffin-embedded Swiss-rolled murine colon tissues using the following antibodies: rabbit polyclonal anti-*C. koseri* (Abcam; 1:50), which cross-reacts with *C. rodentium*, and Alexa Fluor 488-labeled goat anti-rabbit IgG (1:400; Life Technologies) or Alexa Fluor 555-labeled goat anti-rabbit IgG (1:400; Life Technologies) and pseudo-colored green during imaging.^21^ Slides were washed, dried, and mounted using VECTASHIELD HardSet™ Antifade Mounting Medium with DAPI (Fisher Scientific). Fluorescently stained slides were imaged using a Cytation C10 Confocal Imaging Reader and Gen 5+ software (Agilent BioTek).

### Fluorescence Actin Staining (FAS) test

Phalloidin CF488A (Biotium; 1:50) was included in the secondary antibody incubation step of *C. rodentium* immunofluorescence staining and pseudo-colored white during imaging.

### Immunohistochemistry and analysis

Immunoperoxidase staining for Ki-67, myeloperoxidase (MPO), CD3, and cleaved caspase-3 were performed on paraffin-embedded Swiss-rolled murine colon tissues. Sections were deparaffinized, antigens retrieved with citrate buffer, and quenched with H_2_O_2_. Tissues were then incubated overnight at 4°C using the following antibodies: prediluted rabbit polyclonal anti-Ki-67 (Biocare, PRM325AA), prediluted rabbit monoclonal anti-MPO (Biocare, PP023AA), rabbit polyclonal anti-CD3 (Abcam, ab5690; 1:150), or rabbit monoclonal anti-cleaved caspase-3 (Cell Signaling, 9664; 1:400). Primary antibodies were detected with anti-rabbit HRP Polymer (DAKO), color was developed using 3,3′-diaminobenzidine (DAB+), and tissues were counterstained by hematoxylin. All slides were imaged and analyzed using a Cytation C10 Confocal Imaging Reader and Gen 5+ software (Agilent BioTek). Crypt length and the proportion of the crypt that contained Ki-67 positive nuclei were determined by measuring the distance from the base of the crypt to the luminal surface and the last positive nuclei from 3 mid-powered fields, respectively. The average number of CD3-positive cells was quantified by the Cell Analysis function of the Gen 5+ software (Agilent BioTek) and was limited to the mucosa to reduce inclusion of nonspecific staining. The proportion of apoptotic mucosa per 5 high-powered fields was quantified by measuring the total height of the mucosa and the height of the region containing positive cleaved caspase-3 staining.

The average number of MPO-positive cells per 5 high-powered fields was quantified by a gastrointestinal pathologist (MBP) in a blinded manner.

### Colonic lamina propria isolation

Colons were removed, opened longitudinally, washed with cold PBS, cut into 5 mm pieces, and incubated in 50-mL conical tubes containing 25 mL pre-warmed RPMI 1640 media with 5% FBS, 5 mM EDTA, 1 mM dithiothreitol (Thermo Fisher Scientific), and 20 mM HEPES at 37°C for 40 minutes in a non-CO2 MaxQ4450 horizontal shaker (Thermo Fisher Scientific). The media was then strained through a sieve (Everyday Living), and intestinal pieces were placed into 25 mL cold RPMI 1640 media containing 2 mM EDTA, and 20 mM HEPES, shaken vigorously 20 times, and strained again. Intestinal pieces were then minced and placed into 25 mL prewarmed RPMI 1640 media containing 0.1 mg/ml Liberase TL (Roche), 0.05% DNAse I (Sigma-Aldrich) and 20 mM HEPES and shaken at 37°C for 30 minutes. Cells were pulled through a 10 mL syringe 20 times and filtered through a 70 μm cell strainer into an equal volume of cold RPMI 1640 media containing 5% FBS, 0.05% DNAse I, 20 mM HEPES on ice. Cells were spun for 10 minutes at 4°C and 475 × g and resuspended in 40% Percoll (Sigma-Aldrich) solution and underlaid using 90% Percoll. The 40/90 gradient was spun for 25 minutes at 20°C at 475 × *g* with no brake or acceleration applied. The interphase layer was recovered and washed in fluorescence activated cell sorting (FACS) buffer (PBS with 2% FBS and 2 mm EDTA) and spun again for 10 minutes at 20°C and 475 × *g* prior to staining.

### Flow cytometry

For cell surface staining, cells were incubated in the antibody cocktail for 20 minutes at 4°C in the dark. Samples were blocked using 30 μL normal rat serum (StemCell Technologies). Intracellular cytokine staining was performed using Cytofix/Cytoperm (BD) according to the manufacturer’s instructions. Flow cytometric analysis was performed using a 4-Laser Fortessa (BD) with FACSDiva software (BD). Analyses were performed using FlowJo (BD). A live/dead stain (ThermoFisher) was used to only assess live cells. Antibodies used for flow cytometry are listed in Table 2.

**Table 2.** List of flow cytometry antibodies used for this paper.

Antigen-label	Manufacturer	Catalog number
CD45-BV785	Biolegend	103149
CD11b-FITC	Biolegend	101206
Ly6G-APC	Biolegend	127613
CD103-PE	Biolegend	121406
MHCII-PE-Cy7	Biolegend	107629
Ly6C-BV570	Biolegend	128029
Ly6C-PerCPCy5.5	Biolegend	128012
CD11c-BV421	Biolegend	117330
IL-4-PerCP/Cy5.5	Biolegend	504123
IFNgamma-APC	Biolegend	505810
IL-17A-BV421	Biolegend	506926
CD3-PE-Cy7	Biolegend	100320
CD4-APC-Cy7	Biolegend	100414
TNF-FITC	Biolegend	506304
Live/Dead-eFluor506	ThermoFisher	65-0866-14

### Ex vivo T cell stimulation

T cells were stimulated ex vivo with 50 ng/mL phorbol 12-myristate 13-acetate (PMA) and 1 µg/mL ionomycin in the presence of GolgiStop (BD) in T cell media (TCM) (RPMI1640 supplemented with 10% HI-FBS, 1% penicillin/streptomycin (ThermoFisher), 1 mM sodium pyruvate (ThermoFisher), 55 µM 2-mercaptoethanol (ThermoFisher), 1% glutamax (ThermoFisher), and 1% non-essential amino acids (ThermoFisher) for 5 hours.

### Generation of colonoids

Colons were extracted from *Tln1*^*fl/fl*^ and *Tln1*^*Δepi*^ mice, washed with PBS, cut into 5–6 pieces, and incubated in chelating buffer (10 mM EDTA in PBS) at 4°C for 30 min while rocking. The tissues were then vigorously shaken in fresh dissociation buffer (1% w/v D-sorbitol and 1.5% w/v sucrose in PBS) and repeated until a clean fraction of crypts was obtained. The isolated crypts were embedded in Matrigel matrix (Corning, 356231) and maintained in 50% L-WRN conditioned media with 100 U/ml penicillin/streptomycin, 10 μg/ml Gentamicin (Gibco), 10 μM Y27632 (Tocris, 1254), and 10 μM SB431542 (Tocris, 12614). Gentamicin and Y27632 are not included after the first passage.

### Statistics

All the data shown represent the mean ± SEM unless otherwise noted. GraphPad Prism 9.4 (GraphPad Software) was used to perform statistical analyses and significance was set at *P* < 0.05. For normally distributed data, a 2-tailed Student’s *t* test or a 1-way ANOVA with the Tukey or Šídák’s post hoc test were performed to compare differences between two or more test groups, respectively. Non-normally distributed data was analyzed by a 1-way ANOVA with the Kruskal-Wallis test, followed by a Mann-Whitney U test, unless otherwise noted. The Log-rank (Mantel-Cox) test was used to assess differences between the Kaplan-Meier curves of survival. Differences in daily body weights were analyzed by a 2-way ANOVA and Tukey post hoc test.

## Results

### *Tln1*^*Δepi*^ mice have increased susceptibility to *C. rodentium* infection

**Figure 1.**
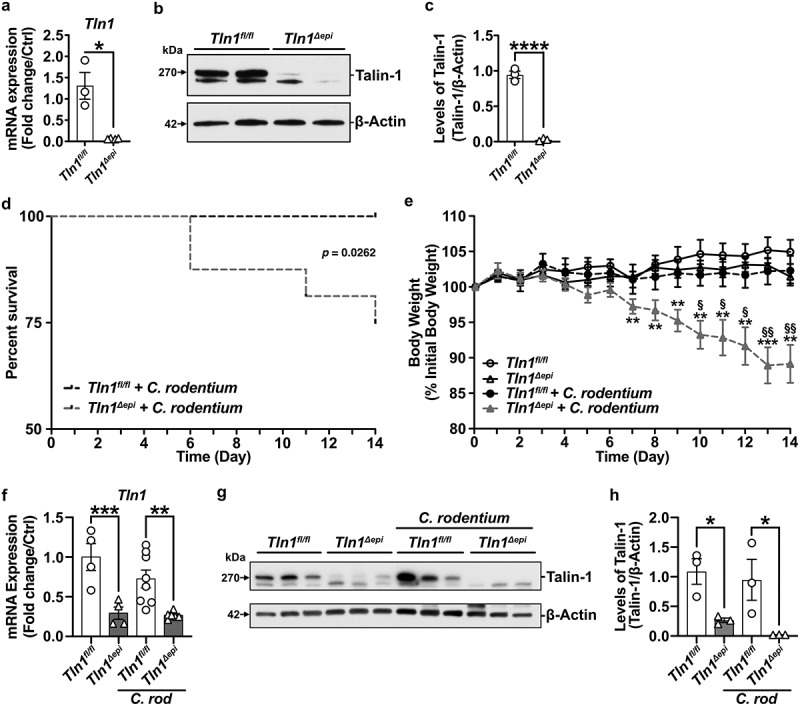
Epithelial-specific deficiency of talin-1 enhances susceptibility to *C. rodentium*-induced death and weight loss. (a-c) Colonic epithelial cells were isolated from *Tln1*^*fl/fl*^ and *Tln1*^*Δepi*^ mice. (a) *Tln1* mRNA expression was determined by RT-qPCR; *n* = 3–4 per genotype. (b) Representative Western blot of talin-1 protein (270 kDa) expression. (c) Densitometry analysis of talin-1 protein expression; *n* = 3 mice per group. (d-f) *Tln1*^*fl/fl*^ and *Tln1*^*Δepi*^ mice were infected with 5 × 10^8^ CFU of *C. rodentium* by oral gavage and monitored daily for 14 days; *n* = 8–9 uninfected mice and *n* = 12–15 infected mice per genotype. Data pooled from 2 independent experiments. (d) Kaplan-Meier curves of uninfected and infected mice. (e) Daily body weights depicted as percent of initial body weight. (f) *Tln1* mRNA expression in whole colon tissues determined by RT-qPCR; *n* = 4 uninfected mice and *n* = 6–8 infected mice per genotype. (g) Western blot of talin-1 protein (270 kDa) and (h) densitometry analysis; *n* = 3 mice per group. All values are reported as mean ± SEM. Statistical analyses, where shown; *P < 0.05, **P < 0.01, ***P < 0.001, and ****P < 0.0001 determined by (a and c) Student’s *t* test, (d) Log-rank (Mantel-Cox) test, (e) 2-way ANOVA and Tukey test, §*P* < 0.05 and ^§§^*P* < 0.01 compared to infected *Tln1*^*fl/fl*^ littermate controls, (f and h) 1-way ANOVA and šídák’s test compared to *Tln1*^*fl/fl*^.

Talin-1 has been implicated in the formation of attaching and effacing lesions in response to EPEC and *C. rodentium*, but this has not been directly studied in vivo.^20,21^ To evaluate the role of talin-1 in epithelial cells during pathogenic colitis, we used *Tln1*^*Δepi*^ and littermate control *Tln1*^*fl/fl*^ mice.^22,24^ We first confirmed knockdown of *Tln1* mRNA (Figure 1a) and talin-1 protein expression in isolated CECs (Figure 1b,c). Next, we inoculated *Tln1*^*Δepi*^ mice and their *Tln1*^*fl/fl*^ littermate controls via oral gavage of 5 × 10^8^ CFUs of *C. rodentium* for 14 days, as we described.^21,27,28^ *Tln1*^*Δepi*^ mice were more susceptible to *C. rodentium*-induced disease, exhibiting decreased survival (Figure 1d) and increased body weight loss (Figure 1e) compared to infected *Tln1*^*fl/fl*^ mice. *Tln1* mRNA and talin-1 protein levels remained significantly reduced in whole tissues of *Tln1*^*Δepi*^ mice with and without infection (Figure 1f-h).

### Epithelial talin-1 contributes to pathogen containment by facilitating actin rearrangement and attachment of *C. rodentium* to the epithelium

**Figure 2.**
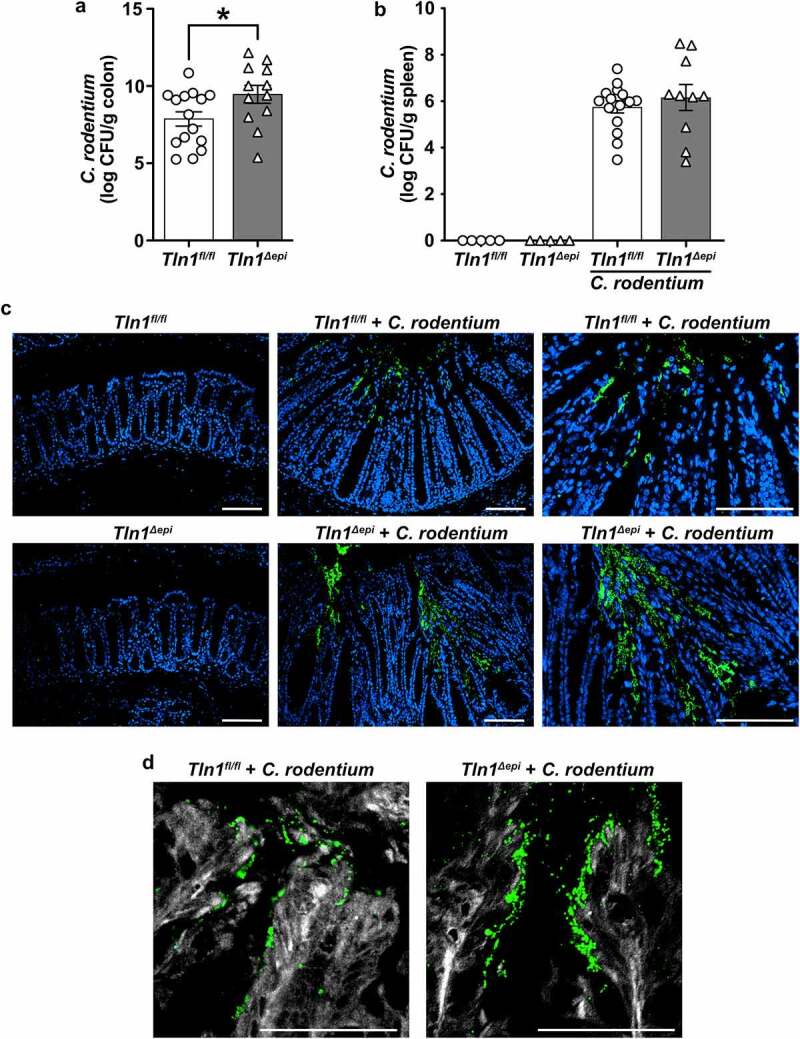
Epithelial-specific talin-1 contributes to pathogen containment by facilitating actin rearrangement and attachment of*C. rodentium* to the epithelium. (a-b) Bacterial burden was assessed by culturing serial dilutions of homogenized tissues and normalizing to tissue weight on day 14 post-infection (p.I.). (a) *C. rodentium* colonization of the colon; *n* = 15 infected *Tln1*^*fl/fl*^ mice and *n* = 12 infected *Tln1*^*Δepi*^ mice. Data pooled from 2 independent experiments. *P < 0.05 determined by Student’s *t* test. (b) *C. rodentium* colonization of the spleen;*n* = 5 uninfected mice per genotype, *n* = 15 infected *Tln1*^*fl/fl*^ mice, and *n* = 10 infected *Tln1*^*Δepi*^ mice. (c) Representative immunofluorescence images of *C. rodentium* (green) and DAPI (blue) in colon tissues of uninfected and infected mice; *n* = 4 mice per group. (d) Representative images of FAS (white) co-stained with *C. rodentium* (green) in colon tissues of uninfected and infected mice; *n* = 4 mice per group. All values are reported as mean ± SEM. Statistical analyses, where shown; *P < 0.05 determined by Student’s *t* test. (c-d) Scale bars represent 100 μm.

It has been suggested that talin-1, a focal adhesion molecule, is necessary for the binding of A/E pathogens and we have reported that diminution of *Tln1* mRNA transcripts results in decreased intimate attachment of *C. rodentium* to colonic epithelial cells in vitro.^7,20,21^ Therefore, we assessed the burden and localization of *C. rodentium* in *Tln1*^*fl/fl*^ and *Tln1*^*Δepi*^ mice infected for 14 days. Mice with epithelial deletion of *Tln1* exhibited a 1.6 log-order increase of *C. rodentium* CFU per gram of colon tissue (Figure 2a). To determine whether the increase in *C. rodentium* colonization in the colon led to increased bacterial dissemination to other organs, we harvested the spleens and assessed viable bacteria. There was no difference between bacterial burden in the spleens of infected *Tln1*^*Δepi*^ mice compared to *Tln1*^*fl/fl*^ mice (Figure 2b). This suggests that epithelial loss of talin-1 does not decrease gut barrier function. Immunofluorescence and confocal microscopy revealed that in *Tln1*^*fl/fl*^ mice, *C. rodentium* was restricted to the apical surface that lines the colon lumen (Figure 2c). In contrast, in mice deficient in epithelial talin-1, the *C. rodentium* extended along the epithelial cells that line the sides of the crypts and into the base (Figure 2c). *C. rodentium*-induced actin polymerization, visualized using FAS and confocal microscopy, was decreased in *Tln1*-deficient epithelial cells (Figure 2d). In addition, detachment of numerous *C. rodentium*-bound epithelial cells was apparent in *Tln1*^*fl/fl*^ mice and this was abolished in the *Tln1*^*Δepi*^ mice (Figure 2d) suggesting that talin-1 is essential for cytoskeletal polymerization and subsequent shedding of compromised epithelial cells.

### Talin-1 moderates *C. rodentium-*induced colitis

**Figure 3.**
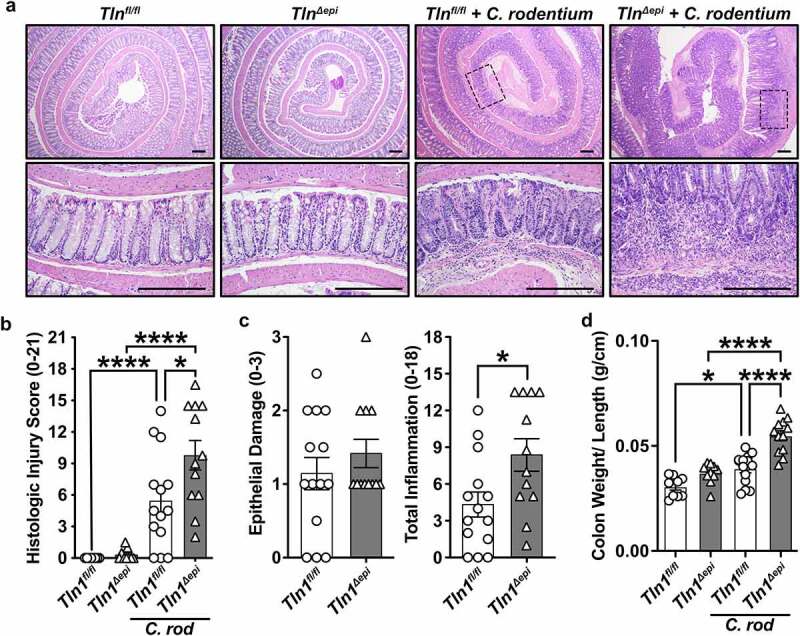
Talin-1 moderates *C. rodentium*-induced acute inflammation. *Tln1*^*fl/fl*^ and *Tln1*^*Δepi*^ mice were infected with 5 × 10^8^ CFU of *C. rodentium* by oral gavage for 14 days. Data pooled from 2 independent experiments. (a) Representative H&E images of the Swiss-rolled colon tissues from uninfected and infected mice. (b) Histologic injury scores derived from the H&E-stained tissues; *n* = 8–9 uninfected mice and *n* = 12–15 infected mice per genotype. (c) Epithelial damage scores and total inflammation scores that were used to generate the histologic injury score in *b*. (d) Colon weight as a proportion of body weight on day 14 post-inoculation. Each symbol is a different mouse. All values are reported as mean ± SEM. Statistical analyses, where shown; *P < 0.05, **P < 0.01, and ****P < 0.0001 determine by (b and d) 1-way ANOVA with Tukey test; (c) Student’s *t* test. (a) Scale bars represent 200 μm.

Animals lacking epithelial *Tln1* exhibited enhanced immune cell infiltration and hyperplasia (Figure 3a), which led to significantly increased histologic injury scores (Figure 3b) compared to *Tln1*^*fl/fl*^ littermate controls infected with *C. rodentium*. The histologic injury score is a composite of the epithelial damage and total inflammation (Figure 3c). While there was exacerbated hyperplasia of the colonic glands (Figure 3c), there was no difference in the score for epithelial damage between infected *Tln1*^*fl/fl*^ and *Tln1*^*Δepi*^ mice, and the increased histologic injury score was driven by a significant increase in total inflammation (Figure 3c). Consistent with the increase in inflammation, the colon weight to length ratio was higher in infected animals and significantly increased in *Tln1*^*Δepi*^ mice compared to *Tln1*^*fl/fl*^ littermate controls (Figure 3d).

## *Tln1* deletion in IECs has no major impact on the composition of the gut microbiota

**Figure 4.**
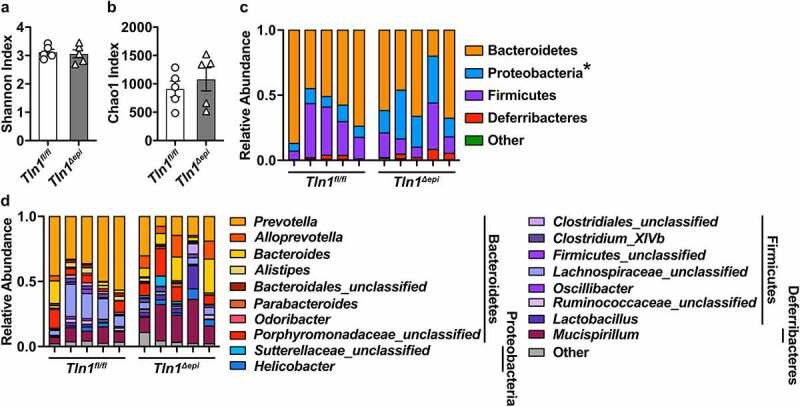
Effect of epithelial talin-1 loss on the fecal microbiome. 16S sequencing was performed on DNA extracted from colonic feces of naïve *Tln1*^*fl/fl*^ and *Tln1*^*Δepi*^ mice; *n* = 5 mice per genotype. Alpha diversity evaluated by (a) Shannon Index and (b) Chao1 Index. The relative abundance of each (c) phylum and (d) genus in the gut colonic bacterial community; *P < 0.05.

Because the composition of the commensal microbiota can regulate colonization by enteropathogens and intestinal inflammation, we analyzed the colonic fecal microbiome from naïve *Tln1*^*fl/fl*^ and *Tln1*^*Δepi*^ mice. Sequencing of the V4 region of the 16S rRNA gene and analysis of the beta diversity revealed no significant differences in the bacterial diversity as determined by the Shannon Index (Figure 4a) or in estimated operational taxonomic units (OTUs) as determined by the Chao1 metric (Figure 4b). As previously reported for C57BL/6 mice,^30,31^ the fecal microbiomes of both *Tln1*^*fl/fl*^ and *Tln1*^*Δepi*^ mice were dominated by the Bacteroidetes and Firmicutes phyla (Figure 4c). The prevalence of Proteobacteria was significantly increased in *Tln1*^*Δepi*^ mice at the phylum level (Figure 4c); however, there were no differences in *Sutterellaceae* or *Helicobacter*, the two main genera belonging to Proteobacteria (Figure 4d). The relative abundance of Bacteroidetes, Firmicutes, and Deferribacteres phyla (Figure 4c), and respective genera (Figure 4d), were similar between *Tln1*^*fl/fl*^ and *Tln1*^*Δepi*^ mice.

### Knockdown of talin-1 in epithelial cells heightens neutrophil recruitment but diminishes the T cell response to pathogenic bacteria

**Figure 5.**
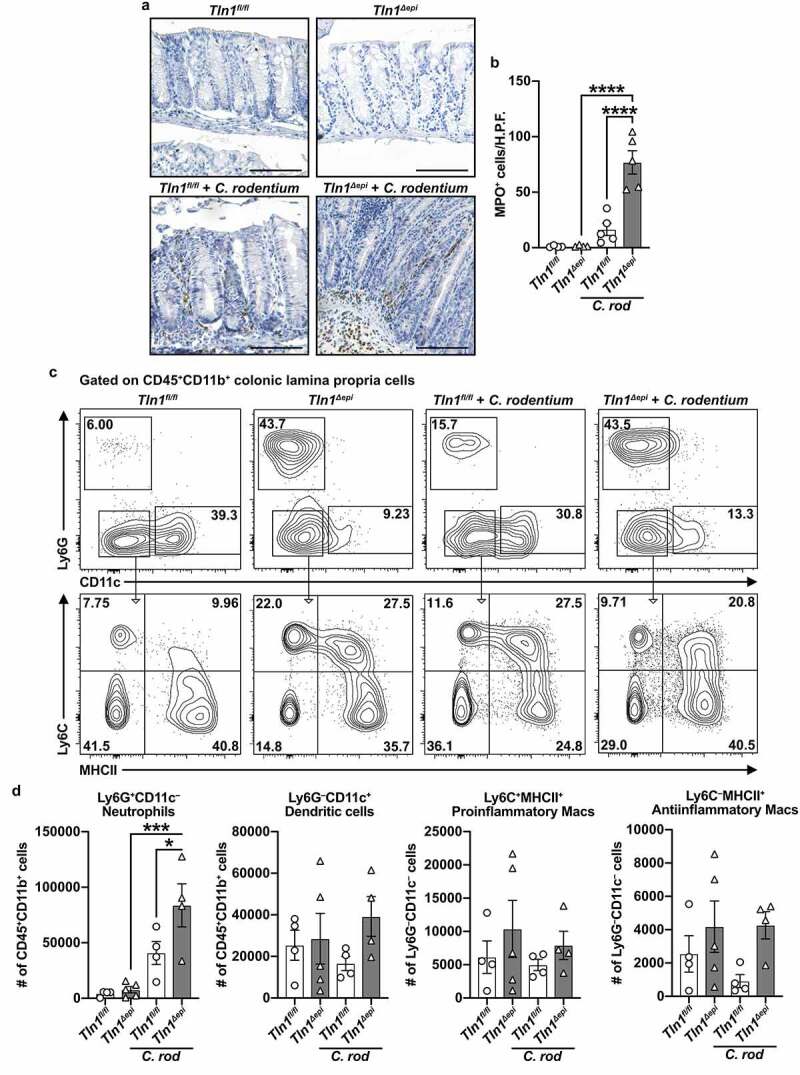
Knockdown of talin-1 in epithelial cells heightens neutrophil recruitment but diminishes the T cell response to pathogenic bacteria. (a) Representative images of colon tissues immunoperoxidase-stained for MPO and (b) the quantification of MPO+ cells per high-powered field (H.P.F). *n* = 4 uninfected mice and *n* = 5 infected mice per genotype. Each symbol is a different mouse. Gating strategy (c) and quantification (d) of myeloid cells isolated from the colonic lamina propria and assessed by flow cytometry; *n* = 4–5 uninfected mice and *n* = 4 infected mice per genotype. Plots were pre-gated for single, live CD45+CD11b+ cells. Neutrophils were identified as CD45+CD11b+Ly6G+CD11c – and dendritic cells as CD45+CD11b+Ly6G – CD11c+. Proinflammatory macrophages were identified as CD45+CD11b+Ly6G – CD11c – Ly6C+MHCII+ and antiinflammatory macrophages as CD45+CD11b+Ly6G – CD11c – Ly6C – MHCII+. All values are reported as mean ± SEM. Statistical analyses, where shown; *P < 0.05, **P < 0.01, and ****P < 0.0001 determined by (b) 1-way ANOVA and Tukey test; (d) 1-way ANOVA and šídák’s test. Scale bars represent 100 μm.

To further evaluate the differences in the inflammatory response between *Tln1*^*fl/fl*^ and *Tln1*^*Δepi*^ mice, we first assessed the immune cell populations by immunohistochemistry. Colon tissues were immunostained for MPO-expressing neutrophils and monocytic cells (Figure 5a). The number of MPO-positive cells was significantly increased in infected *Tln1*^*Δepi*^ mice compared to uninfected *Tln1*^*Δepi*^ mice and infected *Tln1*^*fl/fl*^ mice (Figure 5b). Flow cytometric analysis of CD45+CD11b+ myeloid cells isolated from the colonic lamina propria of *Tln1*^*fl/fl*^ and *Tln1*^*Δepi*^ mice confirmed a significant increase in the number of Ly6G+ neutrophils in infected *Tln1*^*Δepi*^ mice compared to *Tln1*^*Δepi*^ mice and infected *Tln1*^*fl/fl*^ mice (Figure 5c & 5d). We observed no differences in the number of CD11c+ dendritic cells, Ly6C+MHCII+ proinflammatory macrophages, and Ly6C+MHCII – antiinflammatory macrophages between uninfected and infected *Tln1*^*fl/fl*^ and *Tln1*^*Δepi*^ mice (Figures 5c & 5d).

**Figure 6.**
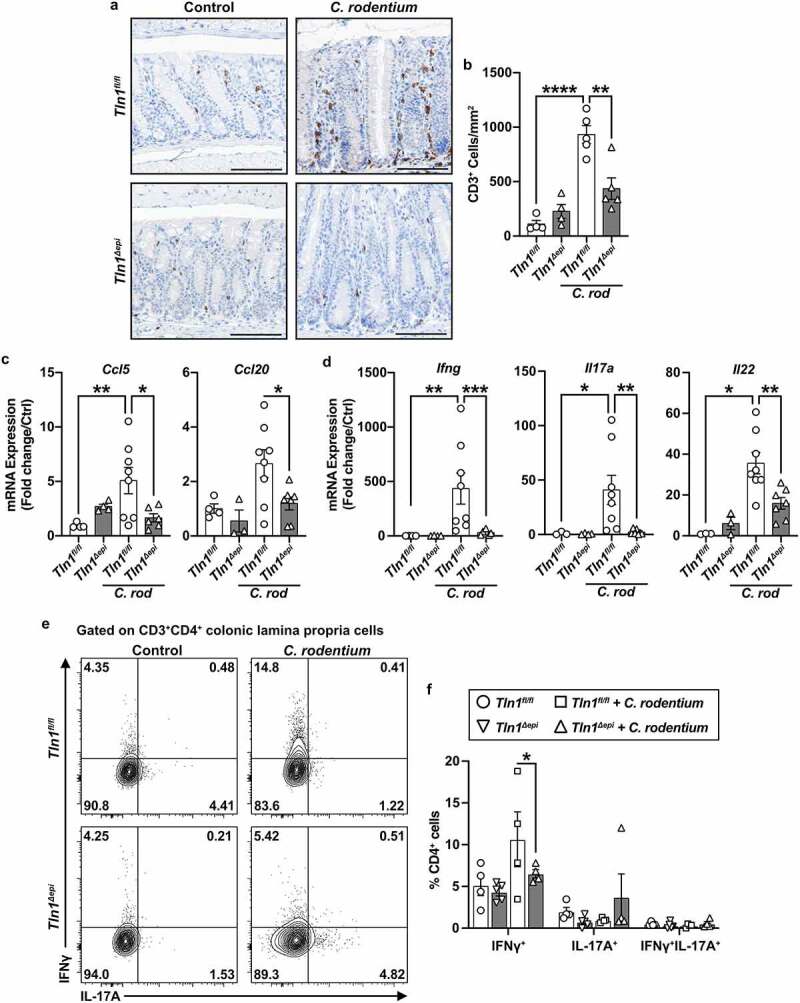
Knockdown of talin-1 in epithelial cells reduces T cell infiltration and activation in the colonic mucosa. (a) Representative images of colon tissues immunoperoxidase-stained for CD3 and (b) the quantification of CD3+ cells per mm^2^. *n* = 4 uninfected mice and *n* = 5 infected mice per genotype. (c) mRNA expression of T cell chemokines analyzed by RT-qPCR. (d) mRNA expression of markers of T cell activation analyzed by RT-qPCR. (c and d) *n* = 4 uninfected mice and *n* = 8 infected mice per genotype. (e and f) Lymphocytes were isolated from the lamina propria of uninfected and infected mice. Following isolation, cells were stimulated with PMA/Ionomycin and GolgiStop, and intracellular cytokine expression was analyzed by flow cytometry. Plots were pre-gated for single, live CD3+CD4+ cells. All values are reported as mean ± SEM. Statistical analyses, where shown; **P < 0.01 and ****P < 0.0001 determined by (b) 1-way ANOVA and Tukey test; (c-d) 1-way ANOVA with Kruskal-Wallis test, followed by a Mann-Whitney *U* test; (f) 1-way ANOVA and šídák’s test. Scale bars represent 100 μm.

In contrast, the elevated number of CD3+ cells in the mucosa of infected *Tln1*^*fl/fl*^ mice was significantly reduced in the tissues of infected *Tln1*^*Δepi*^ mice (Figure 6a,b). Concomitantly, mRNA expression of T cell-attracting chemokines *Ccl5* and *Ccl20* was induced in *Tln1*^*fl/fl*^ mice with infection and was diminished in infected *Tln1*^*Δepi*^ mice (Figure 6c). Additionally, the tissues of *Tln1*^*Δepi*^ mice expressed significantly reduced levels of the transcripts coding for interferon (IFN)-γ, IL-17, and IL-22 (Figure 6d). We then analyzed the production of IFN-γ and IL-17A by CD3+CD4+ T cells isolated from the lamina propria of *Tln1*^*fl/fl*^ and *Tln1*^*Δepi*^ mice infected or not with *C. rodentium* and stimulated with PMA/ionomycin *ex vivo*. We observed an increase in the percent of IFN-γ-expressing T cells in infected *Tln1*^*fl/fl*^ mice that was significantly reduced in *Tln1*^*Δepi*^ mice, but no difference in IL-17A (Figure 6e & 6f). Thus, these data suggest that the role of talin-1 within epithelial cells includes recruitment and activation of T cells, such that when *Tln1* is deleted, there is loss of host defense associated with activated T cells. Additionally, our data suggest that the increased *Il17a* detected in the tissues of the *Tln1*^*Δepi*^ mice most likely derives from other colonic immune cells types than Th cells.

### Loss of epithelial-specific talin-1 enhances pathogen-induced colonic hyperplasia and suppresses epithelial apoptosis

**Figure 7.**
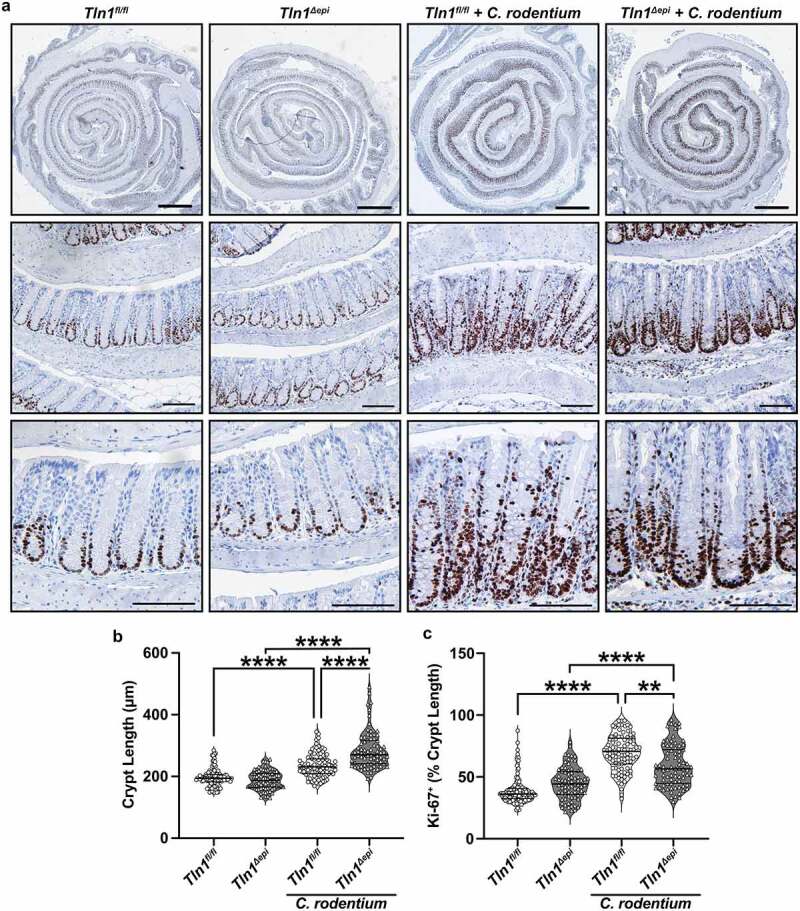
Loss of epithelial-specific talin-1 enhances pathogen-induced colonic hyperplasia. (a) Representative images of colon tissues immunoperoxidase-stained for Ki-67. *n* = 4 uninfected mice and *n* = 4–5 infected mice per genotype. (b) Colonic crypt length. Each dot represents an individual crypt that was visible from base to opening; *n* = 70–107 crypts counted from 4 different mice per group. (c) the proportion of the individual crypts that contained Ki-67+ nuclei determined by measuring from the base of the crypt to the last positive nuclei. All values reported with the median depicted as a thick line and the upper and lower quartiles as thin lines. Statistical analyses, where shown; **P < 0.01 and ****P < 0.0001 determined by 1-way ANOVA and Kruskal-Wallis test. Thick scale bars represent 1000 μm and thin scale bars represent 100 μm.

A hallmark of *C. rodentium* infection is crypt hyperplasia which is characterized by rapid turnover of the epithelial cells lining the crypts and thickening of the colonic mucosa.^5,32^ Therefore, we assessed cellular proliferation in the colonic mucosa of *Tln1*^*fl/fl*^ and *Tln1*^*Δepi*^ mice by immunostaining for Ki-67. *C. rodentium* induced increased Ki-67 expression in both *Tln1*^*fl/fl*^ and *Tln1*^*Δepi*^ mice when compared to uninfected control mice (Figure 7a). In the infected *Tln1*^*fl/fl*^ mice, the proliferating cells extended from the base of the crypt to the luminal surface while in the *Tln1*^*Δepi*^ mice, the positive nuclei did not extend to the lumen and crowded at the base of the crypt (Figure 7a). Infected *Tln1*^*fl/fl*^ and *Tln1*^*Δepi*^ mice had significantly longer crypts compared to uninfected controls and the crypts were further elongated in the infected *Tln1*^*Δepi*^ mice (Figure 7b). Consistent with the photomicrographs of Figure 7a, image analysis confirmed that the proportion of the crypt length in which the Ki-67+ cells extended was significantly reduced in infected *Tln1*^*Δepi*^ mice (Figure 7c).

**Figure 8.**
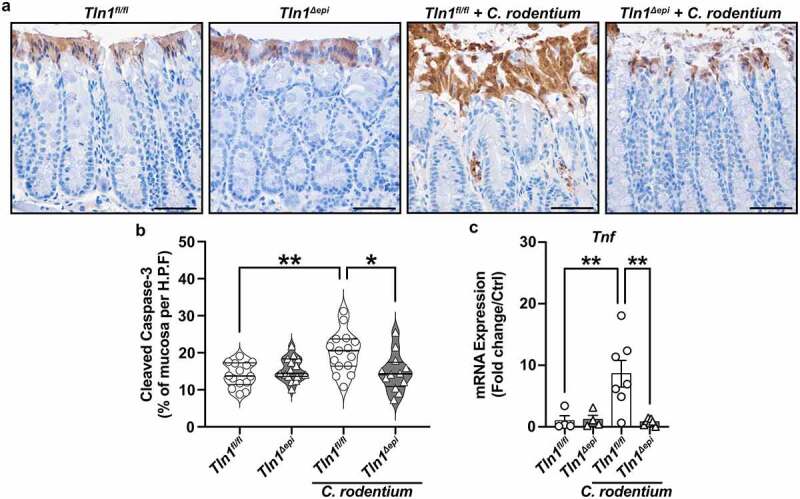
Loss of epithelial-specific talin-1 suppresses pathogen-induced epithelial apoptosis. (a) Representative images of colon tissues immunoperoxidase-stained for cleaved caspase-3. (b) the proportion of cleaved caspase-3-positive mucosa determined by measuring the total height of the mucosa and the height of the region with positive staining. Each dot represents measurements from a high-powered field; *n* = 12–15 fields from 3 different mice per group. All values reported with the median depicted as a thick line and the upper and lower quartiles as thin lines. (c) Expression of the gene encoding TNF-α analyzed by RT-qPCR. *n* = 4 uninfected mice and *n* = 6–7 infected mice per genotype. Each symbol is a different mouse. Values are reported as mean ± SEM. *P < 0.05 and **P < 0.01 determined by (b) 1-way ANOVA and Tukey test and (c) 1-way ANOVA with Kruskal-Wallis test, followed by a Mann-Whitney *U* test. Scale bars represent 100 μm.

To determine the fate of the mature epithelial cells, we assessed apoptosis by immunostaining for cleaved caspase-3. Uninfected mice from each genotype displayed a baseline level of apoptosis that encompassed a single layer of luminal surface cells, which was then increased in *C. rodentium*-infected *Tln1*^*fl/fl*^ mice (Figure 8a). This increase was absent in the *Tln1*^*Δepi*^ mice with infection (Figure 8a), quantified as the proportion of the mucosa containing apoptotic cleaved caspase-3-positive cells (Figure 8b). The mRNA expression of the gene encoding TNF-α, a stimuli of apoptotic cell shedding, was significantly reduced in infected *Tln1*^*Δepi*^ mice compared to infected *Tln1*^*fl/fl*^ mice (Figure 8c).^33^ These data along with the decrease in actin polymerization and shedding of *C. rodentium*-bound cells suggest that talin-1 is important for epithelial cell movement and regeneration in response to challenge, and that this activity is protective during infection.

### Epithelial talin-1 deficiency inhibits epithelial cell mobility

**Figure 9.**
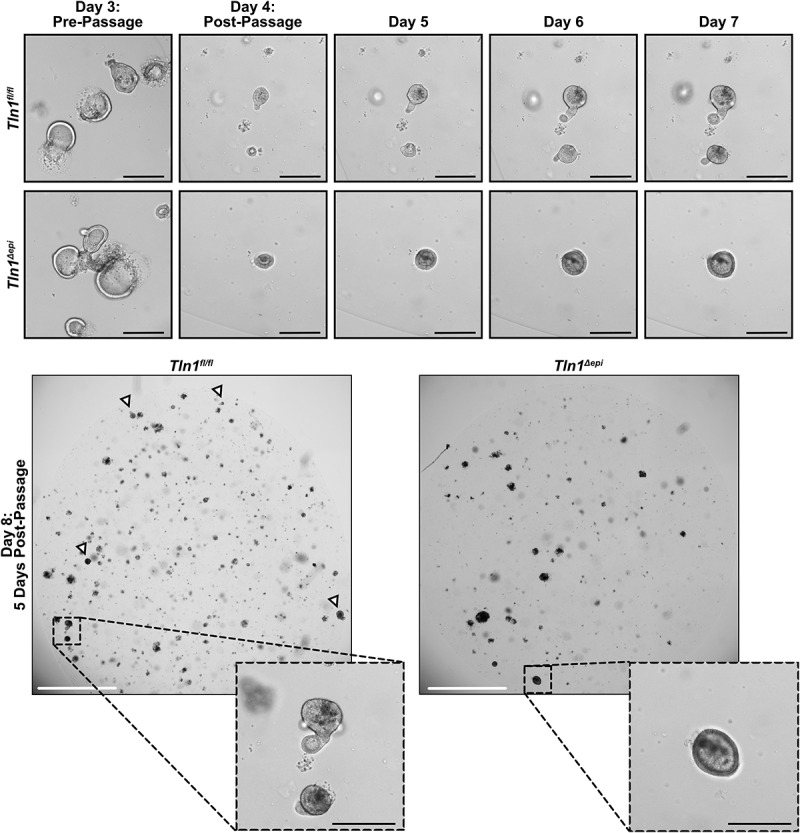
Epithelial talin-1 deficiency inhibits epithelial cell mobility in vitro. Representative images of colon organoids (colonoids) generated from crypts isolated from *Tln1*^*fl/fl*^ and *Tln1*^*Δepi*^ mice and imaged daily for 8 days; *n* = 3 mice per genotype. Arrowheads highlight colonoids with budding. White scale bars represent 2000 μm and black scale bars represent 100 μm.

To assess epithelial motility, we generated 3D organoids (colonoids) from colonic crypts isolated from *Tln1*^*fl/fl*^ and *Tln1*^*Δepi*^ mice. The colonoids were cultured for 3 days, passaged and followed daily. The morphology of the *Tln1*^*fl/fl*^ and *Tln1*^*Δepi*^ colonoids appeared comparable prior to passage (Figure 9). Post-passage, the colonoids from *Tln1*^*fl/fl*^ mice formed irregular structures and buds that protruded into the extracellular growth matrix over time (Figure 9). Conversely, the colonoids derived from *Tln1*^*Δepi*^ mice maintained a uniform spherical shape with minimal to no budding structures and less overall growth (Figure 9).

## Discussion

Talin-1 provides a two-way bridge between the extracellular environment and intracellular networks. Through inside-out signaling, talin-1 induces a conformational change to the integrin heterodimer and increases ligand affinity while also biding to F-actin and vinculin to facilitate focal adhesion assembly and cell migration.^13,15,16,18,34^ Due to the involvement with the cytoskeleton, talin-1 has also been shown to contribute to pedestal formation and actin polymerization in IECs during infection by A/E pathogens in vitro.^20,21^ Thus, we sought to determine if talin-1 is required for bacterial colonization and pathogenesis in the *C. rodentium* mouse model of A/E infection-induced colitis. In this study, we demonstrate that epithelial expression of talin-1 helps contain *C. rodentium* at the luminal surface and protects against mucosal hyperplasia, neutrophil-driven colitis, and severe pathology, including death.

*C. rodentium* shares many of the same virulence factors expressed by EPEC and STEC. One important virulence factor is Tir, which is injected into host cells via the T3SS and triggers actin polymerization following the clustering of bacterial intimin.^5,6^ The N-terminal domain of Tir interacts with host focal adhesion molecules including talin-1, however, this interaction is not necessary for A/E lesion formation as deletion of the N-terminus does not diminish pedestal formation.^6,20,35^ In addition, phosphorylation of the C-terminus of Tir is required for actin condensation, although the translocation of Tir is sufficient for *C. rodentium* colonization, A/E lesion formation, and colonic hyperplasia.^36^ In previous studies, the interaction between bacterial factors and the host cytoskeleton was evaluated using mutant strains. In this study, we directly knocked out an actin binding protein in IECs. Using *C. rodentium*, we observed that loss of talin-1 attenuated actin polymerization, but did not reduce the ability of *C. rodentium* to colonize the colonic mucosa. In fact, the loss of talin-1 enhanced the depth in which *C. rodentium* inhabited the colonic crypts and increased overall colonization. Therefore, we postulate that talin-1 in CECs strengthens the adherence of *C. rodentium* to host cells by enabling actin rearrangement and preventing detachment and movement of the pathogen further into the glands.

*C. rodentium* has adapted multiple mechanisms to hijack the host machinery to increase survival in addition to A/E lesions. A hallmark of *C. rodentium* pathology is transmissible murine crypt hyperplasia.^5,32^ Under homeostatic conditions, colonic epithelial regeneration begins with the proliferation of colonic stem cells followed by maturation of the transit amplifying cells as they migrate up the crypt to the luminal surface. Finally, the terminal cells undergo apoptosis and are shed into the lumen.^37^ This process is accelerated by *C. rodentium* infection and can potentially benefit the bacteria via increased oxygenation of the mucosa as the cells at the apex of the crypts ferment glucose to lactate instead of oxygen.^38^ However, cell extrusion is detrimental to the pathogen as those bacteria attached to the dying cells are shed out into the lumen, which is protective for the host. Not only did we observe a decrease in apoptotic cells at the luminal surface in talin-1-deficient mice, but also crowding of proliferating cells at the base of the crypt with reduced movement of proliferating cells up the sides of the crypts. This resulted in increased crypt elongation that may also contribute to the increased *C. rodentium* colonization. In addition, colonoids derived from *Tln1*^*Δepi*^ mice grew over time, but remained spherical and did not show signs of budding or extension into the ECM substrate. In prior studies of the small intestine, cell proliferation was the primary force that drove enterocyte migration up the villus, a movement that required integrins.^39,40^ Moreover, the relationship of talin-1 with both integrins and actin filaments contributes to cell adhesion and ECM traction for movement.^17,41^ Thus, our data indicates that talin-1 expression in CECs is essential for cell turnover and the movement of proliferating cells up the colonic crypts in a model of infectious colitis.

Interestingly, in addition to the changes we observed in the epithelial cell compartment, the deletion of *Tln1* in CECs also modulated the mucosal immune response. The increased histologic injury scores that *Tln1*^*Δepi*^ mice exhibited was driven by the infiltration of immune cells. *C. rodentium* elicits a robust inflammatory response, recently identified as type 3.^42^ We found that mice lacking epithelial talin-1 displayed higher numbers of MPO+ cells by immunohistochemistry and more neutrophils by flow cytometry in the mucosa, which might seem counterintuitive since there was an increase in bacterial burden. However, mice that do not express TLR4 have decreased recruitment of GR-1+ neutrophils and F4/80+ macrophages to the infected tissues, and are less susceptible to *C. rodentium* pathogenesis, which would be consistent with our findings of increased disease with more infiltration of innate inflammatory cells.^43^ Moreover, in combination with our data, these findings suggest that innate cells are not sufficient to control bacterial growth, and that the presence of *C. rodentium* maintains the pool of MPO-expressing cells in the tissue. Further, immunocompromised *Rag*^*–/–*^ mice do not display the classic signs of *C. rodentium* pathogenesis, but do exhibit impaired bacterial clearance, all of which are reversed with reconstitution of CD4+ cells.^44,45^ In this present study, we observed that CEC-specific deletion of *Tln1* led to decreased T cell recruitment, expression of T cell-attracting chemokines, and expression of the genes encoding IFN-γ, IL-17a, and IL-22. Our findings align with previous studies that found loss of IL-22, and specifically IL-22 expressed by T cells, results in decreased survival, increased weight loss, increased crypt hyperplasia, and increased *C. rodentium* colonization deep into colonic crypts.^46–48^

In conclusion, the results from our study demonstrate that talin-1 plays a pivotal role in host response to infection by *C. rodentium*. Talin-1 expression by CECs not only influences the epithelial compartment, but also affects the immune cell population during bacterial insult. These findings provide insight into the interaction of A/E lesion-forming pathogens with host cell proteins. Contrary to the conventional paradigm that the intimate attachment of A/E pathogens is associated with increased virulence, our data suggest that this process is also to the advantage of the infected host by limiting bacterial invasion of colonic crypts.

## Data Availability

The authors confirm that the data supporting the findings of this study are available within the article. The 16S rRNA sequencing of the fecal microbiota has been deposited on the Sequence Read Archive website with the BioProject ID: PRJNA946899 at this URL: https://www.ncbi.nlm.nih.gov/bioproject/PRJNA946899.
